# Modeling Protective Anti-Tumor Immunity via Preventative Cancer Vaccines Using a Hybrid Agent-based and Delay Differential Equation Approach

**DOI:** 10.1371/journal.pcbi.1002742

**Published:** 2012-10-25

**Authors:** Peter S. Kim, Peter P. Lee

**Affiliations:** 1 School of Mathematics and Statistics, University of Sydney, Sydney, New South Wales, Australia; 2 Cancer Immunotherapeutics and Tumor Immunology, City of Hope and Beckman Research Institute, Duarte, California, United States of America; ETH Zurich, Switzerland

## Abstract

A next generation approach to cancer envisions developing preventative vaccinations to stimulate a person's immune cells, particularly cytotoxic T lymphocytes (CTLs), to eliminate incipient tumors before clinical detection. The purpose of our study is to quantitatively assess whether such an approach would be feasible, and if so, how many anti-cancer CTLs would have to be primed against tumor antigen to provide significant protection. To understand the relevant dynamics, we develop a two-compartment model of tumor-immune interactions at the tumor site and the draining lymph node. We model interactions at the tumor site using an agent-based model (ABM) and dynamics in the lymph node using a system of delay differential equations (DDEs). We combine the models into a hybrid ABM-DDE system and investigate dynamics over a wide range of parameters, including cell proliferation rates, tumor antigenicity, CTL recruitment times, and initial memory CTL populations. Our results indicate that an anti-cancer memory CTL pool of 3% or less can successfully eradicate a tumor population over a wide range of model parameters, implying that a vaccination approach is feasible. In addition, sensitivity analysis of our model reveals conditions that will result in rapid tumor destruction, oscillation, and polynomial rather than exponential decline in the tumor population due to tumor geometry.

## Author Summary

An innovative approach to treating cancer envisions developing preventative anti-cancer vaccines to train a person's immune cells to eliminate early-stage tumors close to genesis. The design of such a treatment strategy requires an understanding of the tumor and immune interactions leading to a successful anti-cancer immune response. To engage this problem, we formulate a mathematical model of the immune response against incipient tumours consisting of as low as hundreds to thousands of cancer cells, which is far below the clinical detection threshold of over 100,000 cells. The model considers the initial stimulation of the immune response and the resulting immune attack on the tumor mass and is formulated as a hybrid agent-based and delay differential equation model. We apply the model to test dynamics over a wide range of dynamic parameters, including immune and tumor cell growth rates and the size of the initial anti-cancer immune population. Our results show that an anti-cancer memory immune cell population of 3% or less can successfully eradicate an incipient tumor population over a wide range of dynamic parameters, indicating that a vaccination approach is feasible.

## Introduction

The most effective way to treat a disease is to prevent its development in the first place. Consequently, a next generation approach to cancer treatment envisions developing preventative cancer vaccines that would train a person's immune response to eliminate tumors near inception by stimulating a person's immune system, especially cytotoxic T lymphocytes (CTLs), to attack cancer cells expressing tumor-associated antigens [1]. Such an immune response would destroy developing tumors close to genesis, before tumor cells have acquired the ability to suppress immune responses or metastasize to other tissues. A successful preventative cancer vaccine would revolutionize the approach to cancer treatment, and several experimental studies have successfully induced CTL responses against different types of tumor cells [2]–[5].

A number of important questions need to be addressed. In particular, is it a realistic goal to immunize a person against cancer, and if so, how many anti-cancer CTLs would be required to provide significant protection against cancer development? There are several conceivable obstacles that could hinder a memory anti-tumor CTL response from being effective. Since cancers develop from colonies of several cells and grow much more gradually than most infectious diseases, developing tumors will only produce a weak antigenic signal, resulting in the activation of only a small fraction of antigen-specific CTLs. Furthermore, activated CTLs will have to encounter the incipient tumor mass in the midst of a large volume of surrounding tissue. It is conceivable that these effects could render an anti-tumor CTL response ineffective. Consequently, the aim of this paper is to assess the feasibility of preventative cancer vaccines from a quantitative perspective.

A challenge to designing effective vaccines will be to understand the quantitative dynamics of the protective anti-tumor CTL response that initiates in the lymph node and proceeds to the tissue containing the tumor. CTL responses almost always begin in lymph nodes rather than the affected tissue. In particular, unactivated CTLs spend most of the time circulating through lymph nodes until they are stimulated by antigen-presenting cells, at which point they proliferate and migrate to the affected tissue [6].

To model this system, we synthesize current experimental research of CTL dynamics into a hybrid mathematical model consisting of a system of delay differential equations (DDEs) and an agent-based model (ABM). Using this hybrid framework, our model connects the fast-timescale dynamics of immune interactions within lymph nodes with the probabilistic, slow-timescale dynamics of immune surveillance in the tumor microenvironment. We then apply the model to investigate rates of tumor elimination under a wide range of parameters, including tumor and CTL proliferation rates, tumor antigenicity, CTL recruitment rates, and initial CTL populations. In addition, the model sheds light on the scale and nature of the dynamics relevant to an immune response against a clinically undetectable, localized microtumor.

Mathematical modeling of tumor growth and tumor immunology has grown rapidly in recent years and several modeling approaches have been applied to understanding these phenomena. For example, a large body of tumor-immune models have been developed using ordinary differential equations (ODEs) [7]–[14] and partial differential equations (PDEs) [15]. (See also [16] for a review of ODE models of tumor-immune interactions and [17], [18] for reviews of ODE and PDE models of tumor growth.) Another approach has focused on agent-based (or cellular automata) models, sometimes coupled with differential equations, to simulate tumor growth [19], [20], tumor growth with angiogenesis [21], [22], and tumor growth in the presence of an immune response [23]–[25]. These models focus primarily on chemotherapy, immunotherapy, and other treatments operating against existing tumors following clinical detection than on protective immunity against undetectable, developing tumors. In our model, we focus on protective anti-tumor immunity by anti-tumor memory CTLs that would be generated by a preventative cancer vaccine.

The ABM-DDE system we develop in the following sections is most similar in formulation to the hybrid cellular automata-PDE model of [24], which also considers host immune responses against growing tumors. However, our model differs from that of [24] in that it simulates interactions in three dimensions rather than two, cell motion and cell contacts take place in Euclidean 3-space rather than on a lattice, and the model simulates two compartments that account for the communication between the tumor site and the lymph node. On the other hand, since the agent-based and cellular automata components of the two models are comparable and simulate tumor populations of similar orders of magnitude (fewer than 100,000 cells), we can readily compare the results and parameter sensitivity analysis of our model with those of [24] as we do in the Results section.

Although our model is formulated in a way that could apply to multiple types of tumors by modifying parameters, a large body of experimental research has been directed toward developing treatment strategies for breast cancer, particularly by identifying potential antigens that could be targeted by preventative breast cancer vaccines [2], [5]. In addition, key model parameters, such as tumor growth rates, are readily available for breast cancer, e.g., [26]–[29], so for the purposes of focusing the scope of our model formulation and parameter sensitivity analysis, we estimate tumor parameters using breast cancer data.

The paper is organized as follows. In the Results, we discuss the results of model simulations. In particular, we show plots of example simulations, conduct a parameter sensitivity analysis, and discuss the conditions under which the tumor population in the ABM could exhibit a polynomial rate of decline, rather than exponential, due to killing by CTLs. In the Discussion, we discuss several natural extensions of the model and directions for future work. In the Models, we present a two-compartment model, consisting of the ABM of the tumor site and the DDE model of the lymph node. We also justify the parameter estimates.

## Results

**Figure 1:**
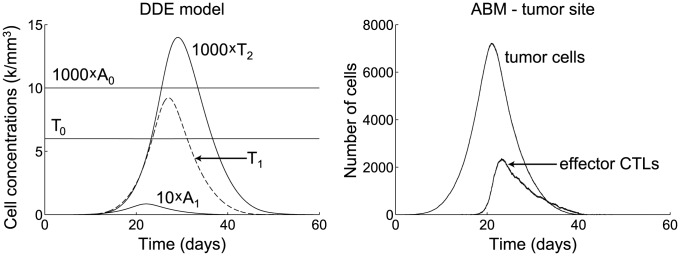
Time plots of cell populations for a simulation of the ABM-DDE system. See also ABM plots in Figure 2. Parameters are taken from the base values shown in Table 1. (left) Numerical solution of the system (4). Populations displayed are 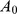, immature APCs in the periphery; 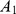, mature APCs in the lymph node; 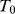 memory CTLs in the lymph node; 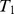, effector CTLs in the lymph node; and 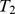, effector CTLs in the periphery. (right) Plot of tumor cell and CTL populations at tumor site.

**Figure 2:**
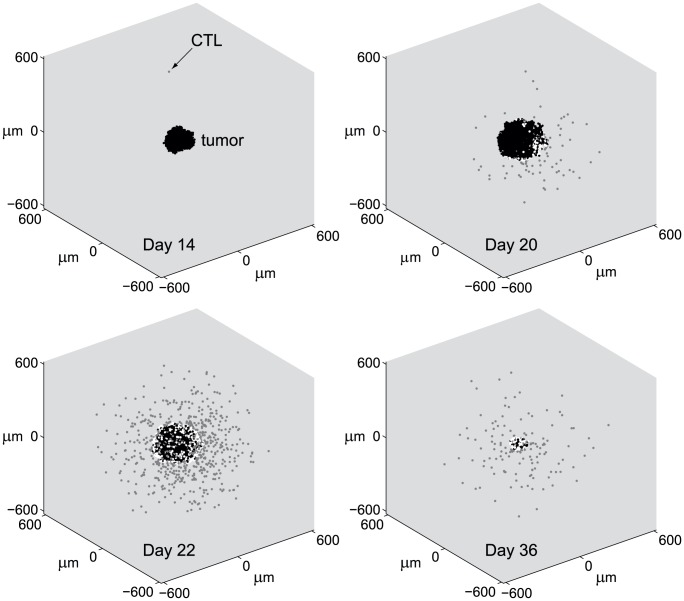
Progression of ABM simulation. See also time plots in Figure 1. Plots show tumor cells (black circles), CTLs that are circulating at the tumor site (gray circles), and CTLs that are engaging tumor cells (white circles). Parameters are taken from the base values shown in Table 1. (Day 14) Tumor grows from one cell to 1,714 cells, triggering a CTL response in the lymph node. CTLs begin circulating in the periphery and occasionally enter the tumor site. (Day 20) Tumor has grown to 6,709 cells. CTLs discover tumor mass, begin to engage tumor cells, and recruit additional CTLs. (Day 22) CTL population overcomes tumor growth causing tumor mass to begin to decrease. Tumor mass is currently 6,880 cells. (Day 36) CTLs continue to engage the tumor, decreasing tumor to 191 cells. All tumor cells are eliminated on day 42.

The hybrid ABM-DDE model was simulated using Matlab R2011b. Results from an example simulation are shown in Figures 1 and 2.

As shown in Figure 1(right), the tumor begins growing in the periphery. As the tumor size reaches approximately 1,000 cells, more and more immature APCs in the periphery become mature, begin presenting tumor antigen, and migrate to the lymph node (see Figure 1(left)). The presence of mature, tumor-antigen-bearing APCs in the lymph node causes memory CTLs to activate into effector CTLs. These effector CTLs proliferate and migrate to the periphery, leading to an anti-tumor CTL response at the tumor site (see Figure 1(right)). Note that only a small fraction of immature APCs and memory CTLs become stimulated into mature APCs and effector CTLs, respectively, so the populations 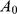 and 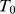 remain almost constant throughout the simulation.

Figure 2 shows snapshots of the ABM simulation at various time points of the CTL response. The tumor begins to grow from one cell at time 0. By day 14, the tumor has grown to 1,714 cells and the anti-tumor CTL response has increased enough so that anti-tumor CTLs begin to circulate around the tumor site at a concentration of 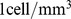. By day 20, several CTLs have engaged the tumor, giving rise to recruitment of additional CTLs. By day 22, the anti-tumor CTL response has overcome tumor growth causing the tumor cell population to decline. By day 36, the tumor has shrunken to 191 cells, and anti-tumor CTLs eliminate all tumor cells on day 42.

**Figure 3:**
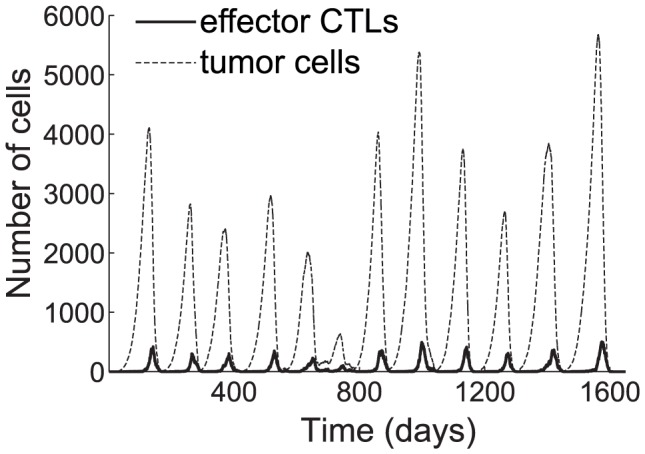
Time plots showing oscillating tumor cell and CTL populations at the tumor site. The average CTL recruitment time, 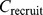, is 24 h. All other parameters are taken from the base values shown in Table 1. The tumor population peaks and declines 12 times. Low points of tumor remissions range from 1 to 105 residual cells. The tumor is eliminated on day 1,600.

Since the system is probabilistic, each simulation produces different results even when the underlying parameters are kept constant. For example, a CTL response will not always eliminate a tumor in one attempt. Indeed, when tumors decrease to tens of cells or fewer, a moderate chance exists that all the CTLs in the vicinity of the shrinking tumor mass may die or migrate away, allowing the residual tumor to relapse. This phenomenon can happen under any set of parameters, but happens more frequently when the average time for CTL recruitment, 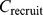, is high (see Figure 3). In Figure 3, the slow rate of CTL recruitment to the tumor site allows the tumor to survive and relapse 11 times. Nonetheless, the memory CTL response keeps the tumor population below 5,700 cells.

These results are corroborated by the cellular automata results of [24], in which Mallet and de Pillis observe that a relatively high CTL recruitment rate leads to few oscillations in the tumor population and early tumor elimination, whereas a lower CTL recruitment rate gives rise to ongoing oscillations, during which the tumor is nearly eliminated at several points, but manages to relapse.

To obtain a broader view of the influence of parameter values on the behavior of the system, we analyze the sensitivity of the model to the following eight parameters: 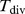, 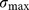, 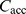, 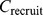, 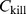, 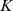, 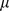, 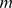, 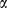. We conduct our sensitivity analysis by varying each parameter individually over the ranges shown in Table 1, while holding all other parameters constant at their base values. For each set of parameters, we conduct 5 simulations. Due to the computational cost of the ABM, we do not conduct more simulations, but even with such few repetitions, we can observe key trends in the influence of the parameters on the model. To assess the influence of the parameters, we calculate the Spearman rank-order correlation of each parameter versus the time to tumor extinction and the maximum number of tumor cells. Table 2 shows Spearman rank-order correlations, 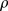, and 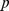-values for each parameter.

**Table 1:** Table of parameters for the ABM and DDE model and estimated values.

Parameter	Description	Estimate (Range)
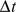	Time step	1 min
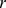	Radius of cells	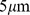
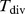	Avg. division time of tumor cell	7 (1–400) days
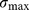	Max unit standard deviation of CTL diffusion	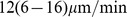
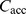	CTL acceleration time from 0 to 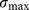	5 (0–24) h
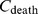	Avg. CTL lifespan	41 h
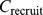	Avg. time for CTL recruitment	8 (2–24) h
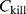	Avg. time for CTL to kill tumor cell	24 (4–48) h
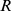	Radius of region of interest	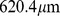
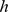	Thickness of CTL cloud	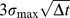
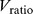	Ratio of volume of tissue to the lymph node	1000
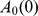	Initial concentration of immature APCs	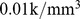
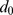	Death/turnover rate of immature APCs	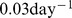
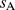	Supply rate of immature APCs	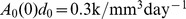
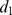	Death/turnover rate of mature APCs	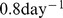
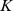	Initial/equilibrium concentration of memory CTLs	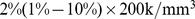
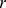	Logistic growth rate of memory CTLs	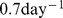
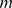	Minimal number of CTL divisions	10 (7–17)
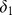	Death/turnover rate of effector CTLs	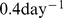
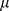	Mass-action coefficient	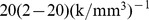 day^−1^
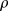	Duration of one CTL division	1/3 day (4–24 h)
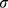	Duration of CTL division program	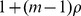
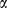	Antigenicity of the tumor	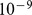 ( 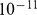 to 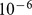 )
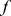	CTLs flow rate out of lymph node to tissue	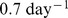

**Table 2:** Spearman rank-order correlations and 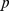-values between model parameters and simulation outcomes.

	Extinction time		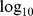 (Max. tumor size)	
Parameter	Correlation 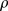	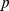 -value	Correlation 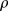	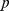 -value
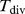	0.9051	1.7506 E-22	−0.8586	3.3583 e-12
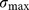	−0.2740	0.1429	−0.6303	0.0002
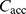	−0.4314	0.0097	−0.3409	0.0451
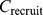	0.7990	1.9968 E-14	0.7661	9.8568 e-13
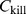	0.2704	0.0366	0.0204	0.8773
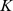	−0.3988	0.0041	−0.4002	0.0040
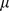	−0.0562	0.6982	−0.3040	0.0319
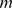	−0.8207	1.7165 E-14	−0.9018	1.1645 e-21
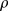	−0.0079	0.9670	0.0259	0.8918
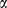	−0.9470	7.9114 E-23	−0.9696	6.1862 e-28

Simulation outcomes are measured in terms of tumor extinction times and maximum tumor populations.

One parameter that stands out as being remarkably insignificant to the final outcome of the simulations is the time-delay parameter, 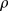, representing the duration of one CTL division. This parameter has almost no correlation to both the time of tumor extinction and the maximum tumor population. One reason for this lack of significance is that over the entire range of 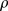, the duration of the CTL division program, 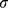, varies from 2.5 to 10 days, while typical tumor extinction times are on the order of 100 days. Hence, a variable delay of several days hardly impacts the final outcome. Similarly, the maximum tumor population will only be minimally affected by a slight delay in the initiation of the CTL response.

Although a natural extension of DDE system is to consider a distributed instead of a discrete time delay for the duration of CTL division, the sensitivity results above imply that a variable delay over the range 4 to 24 hours will probably hardly affect the final outcomes. Moreover, the vast majority of CTLs are likely to have division times within this range [6], [30]

**Figure 4:**
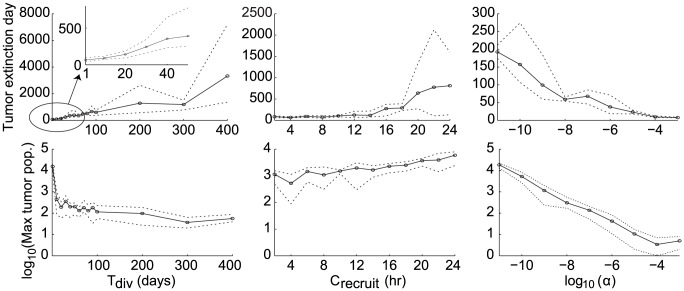
Plots showing tumor extinction times (top row) and 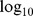 of maximum tumor populations (bottom row) versus parameters, 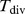, 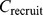, and 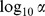 (columns 1, 2, and 3, respectively). Black circles and solid lines represent means over 5 simulations. Dotted lines represent minimum and maximum values obtained over the 5 simulations.

From Table 2, we see that the outcomes of the simulations are most significantly influenced by the average tumor division time, 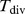; average CTL recruitment time, 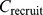; initial number of CTL divisions upon activation, 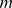; and the antigenicity of the tumor, 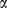. Figure 4 plots the outcomes of the simulations with respect to 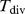, 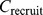, and 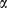.

In column 1 of Figure 4, we see that the time to tumor extinction grows almost linearly with respect to the average tumor division time, 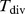. On the other hand, for tumor division times of greater than 10 days, the maximum tumor population hardly changes and nearly all tumors are destroyed at populations of fewer than 1,000 cells. The reason is that the CTL response begins to respond to tumors once they reach a certain size (approximately several hundred cells). A more slowly growing tumor will take proportionally longer to reach this critical size at which CTLs respond. Interestingly, even when an incipient tumor divides at a very rapid rate of once per day, the CTL response destroys the tumor in under 100 days at populations of on the order of 10,000 cells. These results suggest that the immune system responds more effectively to quickly growing tumors. However, very quickly growing tumors can attain orders of magnitude higher populations before being destroyed (see Figure 4 (column 1, bottom row)). As a result, these tumors may grow large and diverse enough to develop immune evasion and metastatic capabilities before the CTL response can eliminate them. On the other hand, tumors that grow very slowly could persist for several years before being detected by CTLs. Consequently, it seems that incipient tumors that grow very quickly or very slowly could cause the most difficulty for an anti-tumor CTL response.

In column 2 of Figure 4, we see that both the time to tumor extinction and the maximum tumor population increase steadily as the average CTL recruitment time, 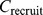, increases. Nonetheless, the maximum tumor population increases by less than an order of magnitude over the range 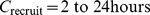. On the other hand, the variance of tumor extinction times seems to increase suddenly once 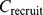 passes 18 hours. This sudden shift is probably due to the increased chance of tumor survival and relapse leading to oscillations when CTL recruitment become sufficiently slow (for example, see Figure 3). This phenomenon may be akin to a Hopf bifurcation in dynamical systems. A useful future direction would be to devise an analogous version of the model as a dynamical system to analyze whether Hopf bifurcations could underlie this and other shifts in the behavior of the ABM-DDE system.

In Table 2, we also see that although the system is sensitive to the CTL recruitment time, it is much less sensitive to the time for CTLs to kill tumor cells. This result is reasonable, since CTLs that ineffectively recruit additional CTLs are unlikely to eliminate the tumor during their lifespans regardless of their killing rate.

In column 3 of Figure 4, we see that tumor antigenicity, 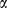, influences the behavior of the system the most. Indeed, a tumor that is 10 times less antigenic than another would require a tenfold higher tumor cell population to elicit a CTL response of the same magnitude. Nonetheless, over the entire simulated range of antigenicities, the CTL response succeeds in destroying the tumor in under than 300 days and at populations below 30,000 cells, corresponding to tumors of less than 0.35 mm in diameter, which is still under the typical clinical detection limit of a few millimeters or greater [31], [32]. Therefore, although it is difficult to estimate the level of antigenicity of an incipient tumor, it appears that an anti-tumor memory CTL response could be reasonably effective for a wide range of tumor antigenicities.

In Table 2, we see that the outcomes of the simulations also depend significantly on 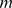, the number of divisions of memory CTLs upon activation. The plots for simulation outcomes versus 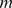 resemble those for 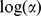 in column 3 of Figure 4, so we do not show them here. The strong dependence of the system on 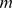 is expected, because each increase or decrease in 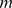 coincides with a twofold increase or decrease in the magnitude of the CTL response. Nonetheless, over the range 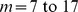, corresponding to a 100 to 100,000-fold CTL expansion upon activation, the maximum tumor populations remain between 5,000 and 100 cells, and extinction times remain under 200 days for all simulations.

**Figure 5:**
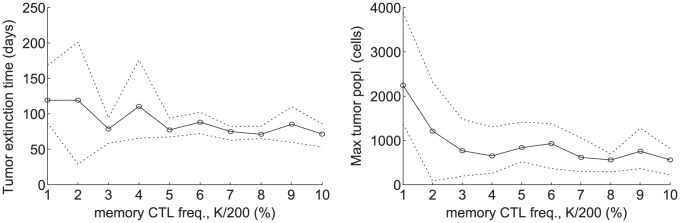
Plots showing tumor extinction times (left) and maximum tumor populations (right) versus frequency of memory CTLs at steady state, i.e. 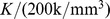. Black circles and solid lines represent means over 5 simulations. Dotted lines represent minimum and maximum values obtained over the 5 simulations.

We also consider the sensitivity of the system to the size of the memory CTL pool, since this parameter will inform the development of a preventative breast-cancer vaccination strategy. Figure 5 shows simulation outcomes against the steady-state frequency of anti-tumor memory CTLs. From the figure, we see that the time to tumor extinction only decreases slightly as the memory CTL population increases. On the other hand, the maximum tumor population decreases approximately threefold as the memory CTL population rises from 1 to 3% and then stabilizes for memory CTL populations from 3 to 10%. Based on this result, a preventative vaccination strategy would maximize its potential efficacy by generating a memory CTL pool of around 2 to 3% of the steady state CTL population. A target memory pool of that size may be attainable by a strategic use of cellular vaccines and adjuvants [33].

The results above indicate that over the range of parameter values considered in Table 1, the presence of anti-tumor memory CTLs effectively restricts the maximum growth and longevity of an incipient tumor, perhaps even to the point of preventing it from diversifying and exhibiting immunosuppressive or metastatic behaviors. As we see in Table 1 most of the varied parameters were considered over a range of at least 1/2 to 2 times the estimated value. The only parameters that were not varied over this wide of a range were the CTL diffusion parameter, 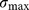, and the minimum number of CTL divisions, 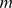, which vary over a range of approximately 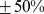 of the estimated value. The ranges of these parameters were tightened, since estimates were based on direct experimental measurements of the required quantities [34]–[36]. In addition, the mass-action coefficient, 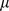, was varied from 1/10 to 1 times the estimated value, since higher values of 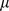 would only make the CTL response stronger and more effective. Consequently, we decide to only consider lower values of this parameter to see whether weaker CTL responses can result in a favorable outcome. From these results, the model suggests that pursuing the development of breast cancer vaccines that would boost immune defenses against incipient tumors may be a feasible preventative treatment strategy over a wide range of parameter values.

### Conditions for tumor escape

In the results above, we do not discuss the probability of tumor survival, because the tumor is always eventually eliminated in our simulations. In fact, for the model as formulated, eventual tumor elimination is highly likely and perhaps guaranteed on an infinite-time horizon.

Several reasons why the model formulation makes eventual tumor elimination very likely are as follows. The modeled tumor site is a finite volume of 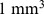, and tumor cells cannot grow beyond this region. CTLs are continually supplied from a regenerating memory population, so CTLs never go extinct. On the other hand, the tumor-free state is an absorbing state from which no new tumor cells can be generated. Over the range of considered model parameters, CTLs proliferate and recruit additional CTLs at a faster rate (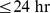) than tumor cells divide (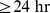), so once CTLs engage tumor cells, the CTL population can always exceed or keep abreast of tumor proliferation. These parameter assumptions seem reasonable, since the expansion rate of proliferating memory CTLs will most likely exceed the growth rate of tumor cells (see the discussion and references in the Parameter Estimates section).

In addition to these considerations, the probabilistic nature of the model implies that there is always a nonzero chance that even a few CTLs can kill a large number of tumor cells rapidly, meaning that a chain of strongly cytotoxic events could lead to complete tumor elimination even in unlikely circumstances. Consequently, over an infinite-time horizon, tumor elimination becomes more likely and perhaps even inevitable. For example, as we see in Figure 3, the underlying dynamics appear to be oscillatory. However, every oscillation increases the chance that the tumor could be eliminated, which occurs on day 1,600 in the displayed simulation.

Due to these limitations in the model, we choose to assess the simulations based on time to tumor elimination and maximum tumor population rather than probability of elimination. Our focus is whether an anti-tumor CTL response can eradicate an incipient tumor quickly and below a certain size, instead of whether it can eventually eliminate the tumor. The reason we are interested in a quick and decisive immune response is that tumors that grow for a long time or to a large size most likely have a higher probability of avoiding immune elimination by either metastasis and migration away from the primary tumor site or by mutating to develop immunosuppressive or immune evasive capabilities. At this point, we do not explicitly model tumor metastasis or adaptive mutation, so these aspects remain a key direction for future work.

As a substitute to directly measuring the probability of tumor elimination, one can set a criterion for failure of the immune response and reinterpret the results. For example, a possible criterion could be that the incipient tumor must be eliminated in fewer than 10,000 tumor cells (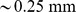 diameter) and in less than 2 years. However, the probability of tumor elimination still follows the same trends shown in Figures 4 and 5, so we do not display additional results under this criterion.

### Rate of tumor decline and elimination

In the simulations above, the tumor decline to extinction appears to follow a curve of the form 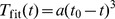, rather than an exponential decay, where 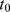 approximately coincides with the simulated extinction time and 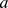 is constant. An interesting observation is that, unlike an exponential decay, the cubic curve 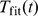 reaches 0 in finite time, meaning that the descent to tumor elimination proceeds almost deterministically, even though the model is probabilistic.

**Figure 6:**
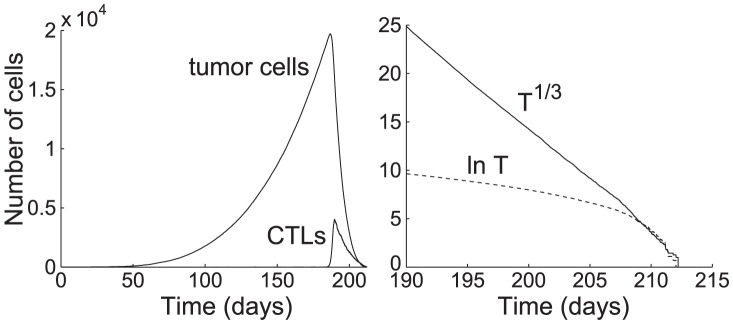
Time plots of tumor and CTL populations from a simulation of the ABM-DDE system. (left) Tumor antigenicity 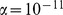. All other parameters are taken from the base values shown in Table 1. The tumor population is extinct on day 213. (right) Plot of the cube root and natural logarithm of the tumor population from day 190 to extinction. The numerical solution of 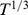 has a high linear correlation 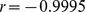, implying that 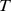 decays as a cubic function of 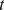. (The linear regression is 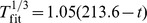.) On the other hand, the numerical solution of 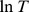 does not exhibit linear behavior, showing that 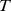 does not decay exponentially.

Figure 6(left) shows the time plots of the tumor and CTL populations from a simulation of the ABM-DDE system, where the tumor antigenicity 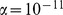 and all other parameters are taken from the base values shown in Table 1. Figure 6(right) shows plots of 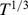 and 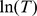 from day 190 to extinction, where 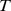 is the tumor population corresponding to the agent-based simulation of the tumor site.

From Figure 6(right), we see that 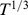 declines almost linearly to the extinction time, whereas 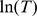 is far from linear, indicating that the tumor decline much more closely follows a cubic function than an exponential decay. The linear regression for 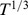 is 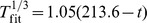. (The plot of the fit is not shown, because it overlaps the curve 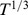 very closely.)

The cubic curve 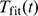 predicts a deterministic finite time extinction of the tumor at time 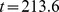, which is very close to the simulated extinction time of 213 days. The cubic decline can be explained by considering the geometry of the system. If we assume that the growing tumor mass is approximately spherical, most CTLs will engage tumor cells on the surface of the sphere. This is not to say that some CTLs will not penetrate the tumor, even causing the tumor to fragment and lose its spherical shape. Indeed, fragmentation happens more frequently as the CTL recruitment rate 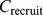 decreases, causing the model to depart from a strictly cubic decline. However, if for the most part, the majority of CTLs encounter and engage the tumor near its surface, the rate that tumor cells are killed by CTLs will be proportional to 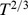. A system that will yield a cubic solution like the one above is a differential equation of the form 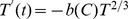, where the coefficient 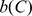 is proportional to how thoroughly CTLs cover the tumor surface and is a function of the total CTL population, 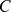.

This observation implies that tumor-CTL dynamics, at least during the decline phase could be modeled by a system of differential equations that predicts deterministic extinction in finite time. In fact, in a different study, a deterministic ODE model of cancer virotherapy was formulated that predicts cancer elimination in finite time [37]. At this point, we leave a more thorough development of a deterministic differential equation model for a future work, but for now, we present the following simple ODE model:
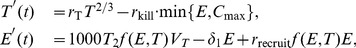
(1)where 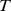 is the number of tumor cells and 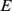 is the number CTLs that are close enough to engage the tumor at its surface. In addition, 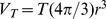 is the tumor volume, where 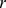 is the cell radius given in Table 1, 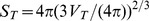 is the tumor surface area, 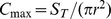 is the maximum number of CTLs that can be in contact with the tumor surface at the current time, and 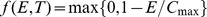 is the density-dependent CTL immigration term.

The first equation in (1) pertains to the number of tumor cells. The first term is the growth rate of the tumor mass. We assume that the growth rate is proportional to the surface area of the tumor, since nearly all growth will happen at or near the surface of the tumor. The second term is the rate that tumor cells are killed by CTLs. The rate of tumor death is proportional to the number of CTLs in contact with the tumor. We assume that all CTLs in the close vicinity of the tumor are in contact with the tumor up to a maximum number 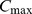. This maximum is the ratio of the surface area of the tumor divided by the cross-sectional area of a CTL.

The second equation in (1) pertains to CTLs in close vicinity of the tumor. The first term is the rate at which CTLs in the periphery come into the close vicinity of the tumor. Since the ODE does not account for CTL diffusion, we assume all CTLs are evenly distributed throughout the periphery at concentration 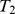, where 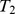 is the CTL concentration in the periphery given by the DDE model (3). Since 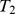 is in units of thousands of cells per mm^3^, the factor 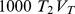 is the number of CTLs that would occupy a region of volume 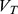. The density-dependent term 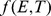 ensures that the rate CTLs come into the vicinity of the tumor decreases to 0 as the population 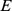 approaches capacity 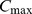. The second term is the death rate of CTLs and the parameter 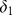 is the same as the one in Table 1. The third term is the rate at which CTLs in the vicinity of the tumor recruit additional CTLs to the vicinity of the tumor. This term is also modified by the density-dependent factor 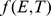 to ensure that the CTL recruitment rate decreases to 0 as the population 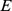 approaches capacity.

**Figure 7:**
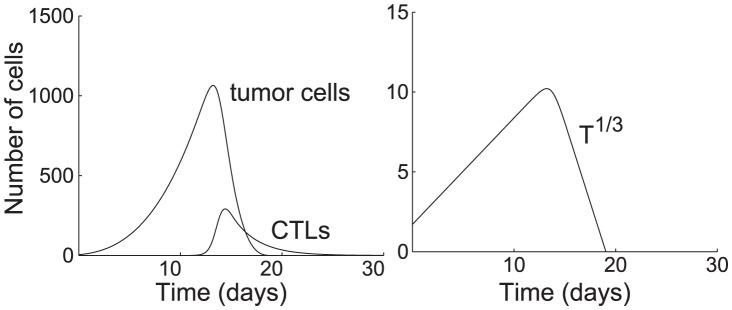
Time plots of tumor population, 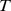, and CTL population, 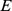, from (1). (left) Parameters 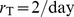, 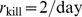, and 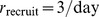. All other parameters are taken from the base values in Table 1. The tumor population is extinct, i.e., identically 0, on day 19.04. (right) Time plot of the cube root of the tumor population. The final decline appears nearly linear.

As with the ABM, the ODE system (1) for the tumor site is coupled with the DDE system (3) for the lymph node. We simulated the combined system using ‘dde23’ in Matlab R2011b. Figure 7(left) shows numerical simulations of the tumor and CTL populations given by (1). Figure7(right) shows a time plot of the cube root of the tumor population obtained from the numerical simulation.

From Figure 7(left), we see that the rise and fall curves of the tumor and CTL populations exhibit similar shapes as those of the ABM-DDE simulation in Figure 6(left). In addition, from Figure 7(right), we see that the final decline of the cube root of the tumor population, 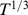, closely follows a linear decline and deterministically reaches extinction in finite time on day 19.04.

These observations suggest that the rate of CTL killing of the tumor resembles a differential equation of the form 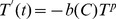 for 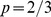, rather than a mass-action model given by 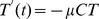. The reason the tumor death rate is proportional to 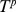 for 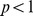 is that not all tumor cells are equally accessed by CTLs due to the geometric structure of the tumor. Because 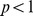, a differential equation model of CTL-tumor dynamics could predict deterministic finite-time extinction of the tumor, and as we see in Figure 6, the dynamics of the ABM could closely follow this deterministic decline to extinction.

## Discussion

We formulate a model of an anti-tumor memory CTL response elicited by vaccination that will act against an incipient tumor. The primary goal of the model is to assess whether it is realistic for a person's immune system to have a sufficient pool of anti-cancer memory CTLs to significantly reduce the chances of developing cancer. We focus our investigation on breast cancer, since extensive experimental research has been done on growth parameters and tumor sizes, e.g., in [26]–[29] and clinical detection limits, e.g., in [31], [32].

Our model suggests that protective immunity against the development of breast cancer could be feasible, because an anti-tumor memory CTL pool of 3% of CTLs could eliminate a developing tumor before it reaches an average size of 1,000 cells, and an anti-tumor memory CTL pool of only 1% of CTLs could eliminate a growing tumor in fewer than 30,000 cells (a diameter of approximately 0.35 mm). These predictions are corroborated by experimental results. In one mouse study, vaccination with telomerase led to telomerase-specific T cell responses of no more than 3% in different mouse strains and had a protective effect against tumor growth [38], and another mouse model showed that a 2% threshold for a vaccine-elicited T cell response predicted efficacy in limiting tumor growth and survival [39].

In our simulations, the effectiveness of the anti-tumor CTL response depends largely on how quickly CTLs can locate and then eliminate an incipient tumor. The key challenges to locating the tumor are that the incipient tumor expresses a very low antigenic signal to the draining lymph node and it takes up a tiny volume in the tissue. The rapidity of this phase depends primarily on the number of CTLs that become activated and migrate to the periphery. These dynamics are governed mostly by 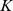, the equilibrium memory CTL population; 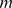, the number of divisions undertaken by an activated CTL; and 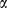, the antigenicity of the tumor. Once the tumor has been located and CTLs begin to engage tumor cells, the survival of the tumor depends mostly on 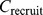, the rate additional CTLs are recruited to the tumor site and, to a slightly lesser degree, 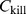, the rate at which CTLs kill tumor cells. A future step for experimental and modeling research will be to understand how to design an optimal vaccination strategy that would elicit a sufficient CTL response to seed an adequate anti-tumor memory CTL pool [33].

An additional observation from our simulations is that faster growing tumors are often destroyed faster than more slowly growing ones. This result agrees with experimental observations that CTL responses react more effectively to rapidly increasing sources of antigen than to constant or slowly increasing stimuli [40]. In other words, for a protective immune response, a population of rapidly growing tumor cells might not be more difficult for the immune response to eliminate than a very slowly growing population.

In our current study, we are interested conditions that allow the CTL response to eliminate a tumor before it reaches a sufficient size or diversity to effectively suppress the immune response, metastasize, or induce angiogenesis. The question remains: At what size or in what time frame is a tumor likely to develop these capabilities, and how would this development impact the immune response. Thus, a direction for future work would be to incorporate the mutation of tumors cells to model the competition between the CTL response and the evolution of the tumor cell population.

Another extension of the model is to explicitly incorporate the chemotaxis of CTLs up a signal gradient to the tumor site. We currently model CTL recruitment using an approach analogous to that of [24], in which new CTLs arrive at a probabilistic rate in the vicinity of recruiting CTLs. In reality, CTLs migrate up a signal gradient toward a region of high cytotoxic activity. However, this process appears to happen much more quickly than the time scale of the CTL response simulated in the model [3]. As a result, in this study, we do not explicitly model the trajectory of recruited CTLs toward the tumor mass. Indeed, if CTLs move at an average rate of 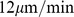, and the radius of the simulated tumor site is 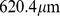, a migrating CTL could travel from the boundary of the region to the center in less than an hour.

As discussed at the beginning of this paper, various models have recently been developed for immune interactions with solid tumors, using both probabilistic agent-based (or cellular automata), deterministic differential equation, and hybrid approaches, e.g., [7], [8], [12], [23], [24]. A future step will be to bridge these frameworks. In the case of our ABM, we noticed that the decline in the tumor population could closely follow a cubic rather than an exponential curve. Consequently, a deterministic differential equation version of the ABM would have to account for the tumor geometry as well as cell localization around the tumor. Developing a differential equation version of the ABM will provide a means of analyzing the stability of the system, particularly around the tumor-free fixed point and determining what conditions allow the tumor to be eliminated in finite time, see [37]. In addition, a differential equation model will shed light on whether a stability bifurcation underlies the rapid increase in the amplitude of oscillations that occur as the CTL recruitment time increases (see Figure 4(column 2)).

Characterizing tumor-immune dynamics using different modeling perspectives will provide a means of assessing whether it would be feasible to prevent breast cancer using preventative vaccines. Since nearly all relevant cell interactions for protective anti-tumor immunity occur at a level below clinical detection, insights provided by models of immune responses against developing tumors will inform further modeling and experimental directions and aid the advancement of next-generation therapeutic strategies.

## Models

Our model considers two compartments of immune activity: the site of the incipient tumor in the tissue and a tumor-draining lymph node. We model dynamics of the tumor compartment using a probabilistic ABM. The advantage of the ABM is that it allows us to capture the probabilistic nature and spatial structure of CTL-tumor interactions. In our simulations, cell populations fall under 100,000, making an ABM computationally practical.

On the other hand, we model dynamics in the lymph node using a system of DDEs. The advantage of the DDE system is that it allows us to capture the dynamics of an arbitrary number of cells efficiently. In the lymph node, immune cells interact at a faster time scale and exist at orders of magnitude higher concentrations than in the periphery, making an ABM formulation computationally impractical. As a result, we devise a hybrid model connecting an ABM and a DDE system for the tumor site and lymph node.

### Agent-based model of dynamics at tumor site

The ABM simulates tumor cells and CTLs at the tumor site. All cells are modeled as spheres of radius 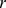 in Euclidean 3-space, and no two cells can overlap the same space. The system is updated according to algorithmic rules at discrete time steps 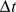. The rules for each type of cell are described below.

#### Tumor cells

**Figure 8:**
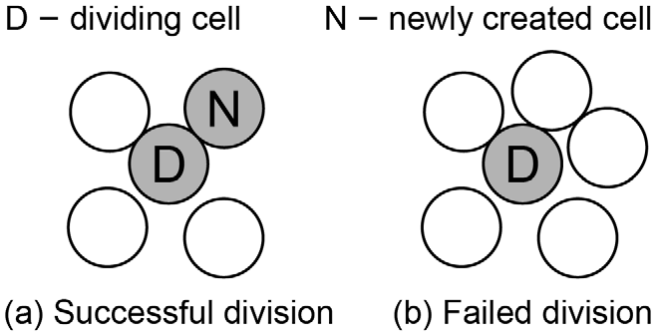
Possible outcomes of cell division. (a) Space is available, so the cell divides successfully, and a new cell is generated adjacent to the old cell. (b) No space is available, so the cell fails to divide. To simplify diagrams, figures are shown in 2-D, although the model occurs in 3-D. package.

At every time step, each tumor cell divides with probability 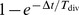, where 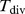 is the average division time of a tumor cell. When a cell divides, it attempts to create a new cell tangent to the original one. The location of the new cell is chosen uniformly at random among all directions that are not currently occupied by an existing tumor cell or CTL. If there is no space for a new cell to appear, the dividing cell fails to divide, and no new cell is created. Figure 8 shows examples of what could happen when a cell attempts to divide.

**Figure 9:**
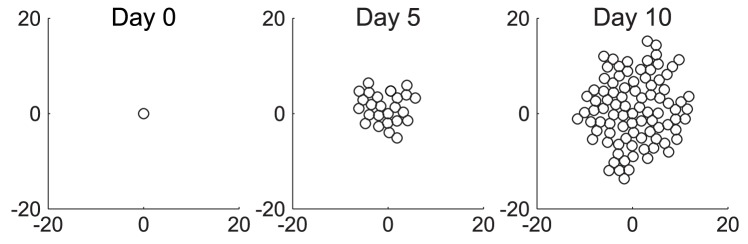
Plots of tumor cells growing from one cell at days 0, 5, and 10. The average time between cell divisions 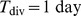. To simplify diagrams, figures are shown in 2-D, although the model occurs in 3-D.

In this model, cancer cells do not move, so a tumor only grows by division, rather than migration. Since our primary focus is to model CTL responses against clinically undetectable microtumors, we assume cancer cells have not acquired the ability to migrate at this stage. In addition, for simplicity, we do not model the non-cancerous tissue surrounding the tumor. Instead, we assume that each new tumor cell can push away the surrounding tissue as it proliferates. Figure 9 shows an example of a growing tumor at various time points. If a cancer cell is killed by an anti-tumor CTL, it is removed from the system.

#### Anti-cancer CTLs

**Figure 10:**
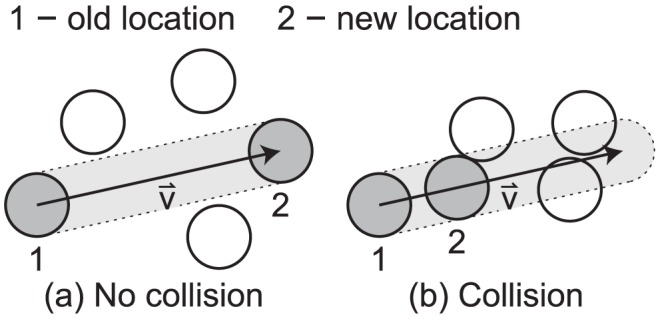
When CTLs move during a time step, two scenarios may occur. (a) No collision – the CTL moves the entire length of 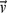, (b) Collision – the CTL moves as far as possible without colliding with another cell.

Unlike cancer cells, anti-cancer CTLs continually move, and we model their movement using a 3-D Wiener process. At each time step 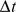, a CTLs location changes by the vector 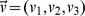, where each coordinate 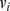 is an independent random variable with normal distribution 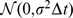 and 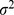 is the variance per unit time of the particle's motion. Note that if 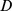 is the diffusion rate of the CTL, then 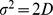 [41], [42]. If a CTL's motion causes it to collide with another cell, the CTL moves as far as possible and stops. See Figure 10.

A CTL that is in contact with a cancer cell stops moving and engages the cancer cell. Then, at each time step, the CTL may perform two possible actions: (1) recruit an additional CTL or (2) kill the cancer cell.

We model CTL recruitment in a fashion similar to that used in the celluar automata model of Mallet *et al.* [24]. Specifically, when a CTL recruits another CTL, a new CTL appears at a location adjacent to the recruiting CTL. A CTL that is engaging a cancer cell recruit an additional CTL with probability 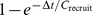 at each time step, where 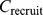 is the average time recruitment time. As in cancer cell division, the direction of the new CTL is chosen uniformly at random among all directions that are not currently occupied by other cells.

This modeling approach is an approximation of CTL chemotaxis along a chemokine gradient toward a site of CTL stimulation [43], [44]. The approximation does not account for the actual trajectory of recruited CTLs toward the recruiting CTL. Instead, the model assumes that CTLs recruited in this fashion originate in the region beyond the tumor site and end up in the vicinity of the recruiting CTL at an average rate, 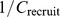. As in [24], this process CTL recruitment is separate and independent of the diffusive random walks (governed by 3-D Wiener processes) of other CTLs within the modeled region of the tumor site. In this manner, CTLs arrive in the vicinity of the tumor either through recruitment by CTLs already engaging cancer cells or by undirected diffusion toward the tumor via a 3-D random walk.

In addition to recruitment, at each time step, a CTL engaging a cancer cell can kill the cancer cell with probability 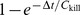, where 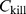 are the average time for a CTL or kill a cancer cell. When a cancer cell dies, any CTLs engaging that cancer cell automatically disengage and begin moving again.

When a CTL starts moving, it accelerates up to the maximum unit standard deviation 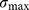. We model CTL acceleration as
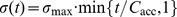
(2)where 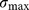 is the maximum unit standard deviation of a CTL and 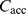 is the time required to accelerate from stationary to the maximum diffusion rate. A justification for this expression is given in the subsection on CTL acceleration.

**Figure 11:**
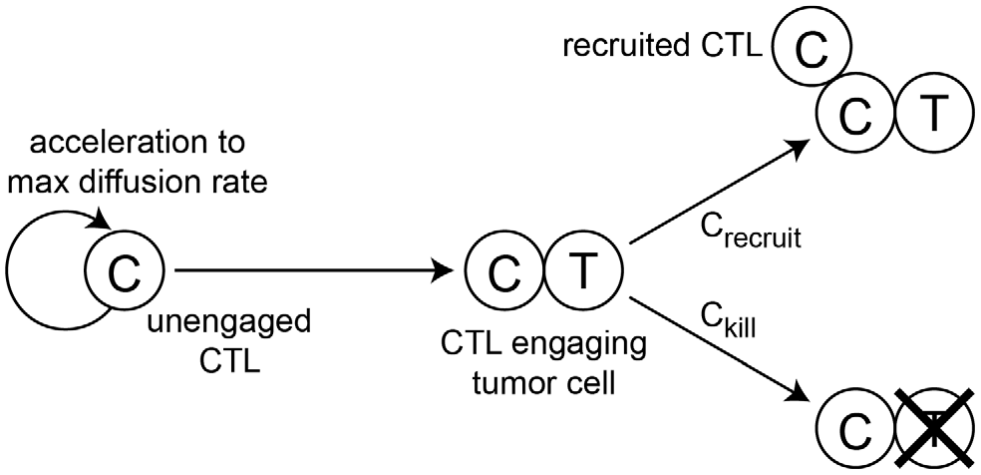
Possible CTL actions during a time step. At each time step, CTLs move according to a 3-D Wiener process. A CTL in contact with a cancer cell stops moving and engages the cancer cell. A CTL engaging cancer cell may recruit an additional anti-cancer CTL with probability 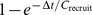 or kill the cancer cell with probability 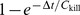. When the cancer cell dies, the CTL disengages and accelerates up to the maximum rate. Although not shown, all CTLs may die with probability 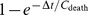.

At each time step, CTLs die with probability 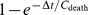, where 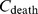 is the average CTL lifespan. Cancer cells and CTLs that die are removed from the system. See Figure 11 for a diagram of possible CTL actions during a time step.

For practicality, we simulate CTL-cancer dynamics in a spherical region of radius 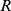 rather than over the entire body. As a result, we must consider CTL immigration and emigration from the region of interest. Outside the spherical region, we assume the CTLs exist at a concentration 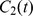 that may vary over time. The value of 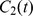 over time is governed by the DDE model.

**Figure 12:**
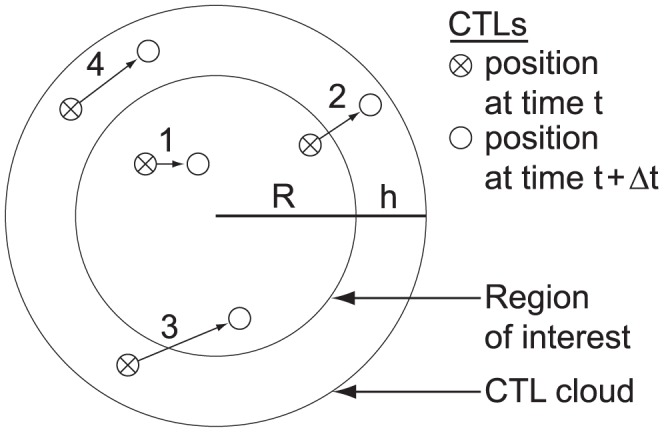
Model of CTL migration between the region of interest of radius 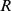 and the CTL cloud of thickness 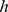. In this example, two CTLs already exist in the region of interest, i.e., 1 and 2. At the beginning of the time step, new CTLs are randomly generated in the CTL cloud, i.e., 3 and 4. CTL motion is calculated for the next time step, and at the end of the time step, CTLs beyond the region of interest are eliminated, i.e., 2 and 4, while CTLs in the region of interest are retained, i.e., 1 and 3.

Once we obtain 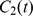 from the DDE model, we consider an annulus of thickness 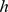 around the spherical region, which we call the *CTL cloud*. Then, at the beginning of each time step, we generate a random number of CTLs distributed uniformly at random throughout the CTL cloud so that no two cells overlap. The number of CTLs generated is given by a Poisson random variable with Poisson parameter 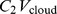, where 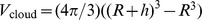 is the volume of the CTL cloud. Next, we update the motion of all CTLs during the time step and only keep CTLs that are inside the region of interest at the end of the time step. We assume all CTLs originating in the cloud diffuse at the maximum rate. See Figure 12.

The CTL cloud represents the region of CTLs that could emigrate into the region of interest during the next time step. To reliably capture nearly all such CTLs, we need to set the width 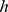 of the cloud high enough such that there is low probability that a CTL on the outside of the cloud could cross over the cloud into the region of interest within one time step. This approach of simulating CTL emigration is our discrete, probabilistic analog of setting a constant boundary condition for a PDE.

### Delay differential equation model of lymph node dynamics

**Figure 13:**
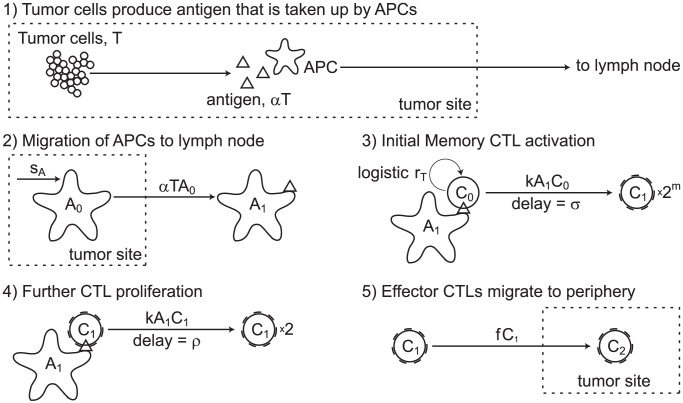
Model of dynamics in the lymph node. (1) Tumor cells produce antigen at the tumor site. (2) APCs pick up tumor antigen, mature, and migrate to the lymph node. (3) Mature antigen-bearing APCs present antigen to memory CTLs causing them to activate and enter the division program of 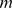 divisions. (4) Effector CTLs that have completed the division program continue to divide upon further interaction with mature, antigen-bearing APCs. (5) Effector CTLs continually migrate to the periphery. Although not indicated, each cell in the diagram also has a natural death rate.

To investigate the possible strength of a secondary anti-cancer CTL response, we simulate the anti-cancer immune dynamics in a vaccinated host. The anti-tumor CTL response begins when antigen presenting cells (APCs) bearing tumor antigen mature and migrate to the draining lymph node, where they activate memory CTLs that begin to proliferate and emigrate to the site of infection. We model this process in five steps illustrated in Figure 13:

1. Tumor cells produce antigen at the tumor site,
2. APCs pick up tumor antigen and migrate to the draining lymph node,
3. In the lymph node, mature APCs activate memory CTLs that enter a minimal division program of 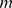 cell divisions,
4. Memory CTLs that have completed the minimal division program become effector CTLs that continue dividing upon further stimulation by APCs,
5. Effector CTLs continually migrate out of the lymph node to the periphery.

The model is formulated as the following system of DDEs:
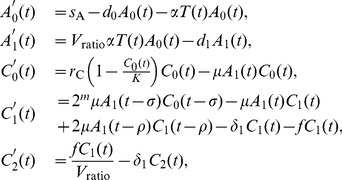
(3)where 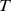 is the tumor cell population at the tumor site modeled by the ABM, 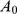 is the concentration of APCs in the periphery, 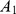 is the concentration of APCs that have matured, started to present tumor antigen, and migrated to the lymph node, 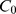 is the concentration of memory CTLs in the lymph node, 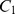 is the concentration of effector CTLs in the lymph node, and 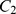 is the concentration of effector CTLs in the periphery. (The concentration 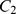 is the value used by the ABM to generate CTLs in the CTL cloud.) Concentrations are measured in units of 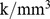 (thousands of cells per cubic millimeter). Note that 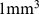 = 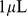 (microliter).

The first equation in (3) pertains to APCs waiting in the periphery. These cells are supplied at a constant rate, 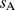, and die at a proportional rate, 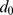. Thus, without stimulation, the population remains at its equilibrium level, 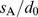. The factor 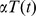 is the proportional rate that APCs take up tumor antigen, mature, and migrate to the lymph node. Rather than explicitly modeling antigen generation, we assume that the rate of APC stimulation is proportional to the tumor population, 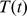, where 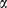 is a constant related to the antigenicity of the tumor.

The second equation in (3) pertains to APCs that have matured, started to present tumor antigen, and migrated to the lymph node. The model accounts for APC maturation, antigen presentation, and migration as one collective event, because APCs that only undergo one or two of the three processes are not pertinent to the dynamics of the model, since they cannot stimulate tumor-specific CTLs. The first term of the equation corresponds to the rate at which these APCs enter the lymph node. The factor 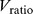 is the ratio between the volumes of the tissue and the draining lymph node. Since we measure populations in terms of concentration, this factor is necessary to account for the change in concentration due to traveling between regions of different volume. The second term is the natural death rate of population.

The third equation in (3) pertains to memory CTLs in the lymph node. The population is replenished up to an equilibrium capacity, 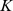, according to a logistic growth model with rate 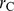. The second term is the rate of stimulation by mature APCs. The bilinear form of this term follows the law of mass action where 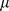 is the proportionality constant, or mass-action coefficient.

The fourth equation in (3) pertains to effector CTLs that have finished the division program of 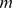 divisions. The first term gives the rate at which activated memory CTLs enter the effector state, 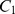. This term corresponds to the final term of the previous equation for 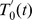, except that it has an additional coefficient of 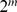 and it depends on cell concentrations at time 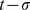. The coefficient 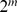 accounts for the increase in population of memory CTLs after 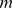 divisions, and the time delay, 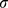, is the duration of the division program. This term accounts for newly proliferated effector CTLs that appear in the 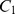 population 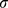 time units after activation from 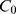. The second term is the rate at which 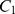 cells are stimulated by mature APCs for further division and the third term is the rate at which dividing cells reenter the system 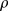 time units later after undergoing one cell division. The time delay 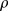 is the duration of one cell division. The fourth term corresponds to the death of 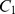 cells at rate 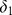. The last term is the rate at which effector CTLs flow out of the lymph node to the tissue at rate 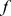.

The last equation in (3) pertains to effector CTLs in the tissue. The first term is the rate at which effector CTLs in the lymph node flow out to the tissue. As with the inflow rate of APCs into the lymph node, this term is scaled by the volume ratio 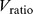. As shown in the last term, effector CTLs in the tissue die at the same rate at effector CTLs in the lymph node.

To incorporate the model (3) with the ABM, we translate the DDE system (3) into a system of difference equations evaluated at time steps of length 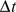, the same time step for the ABM. Our derivation of a system of difference equations from the continuous system is comparable to the reverse process of that used in [45] to translate an agent-based model to a partial differential equation system. More precisely, we translate the system from DDEs to difference equations by assuming that the population variables are constant over intervals of length 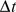 and that the rates of state transitions across time steps are governed by Poisson processes.

However, since the lymph node contains orders of magnitude higher concentrations of immune cells than the tissue [46], [47] and hence interactions occur orders of magnitude more rapidly [48], we additionally assume that (1) immune populations in lymph node are continuous and (2) transition rates governed by Poisson processes closely follow the mean field rates. In other words, instead of using Poisson random variables 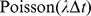, we model transition rates using deterministic factors of the form 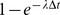. As a result, we do not consider stochasticity or discrete populations in the lymph node.

Furthermore, we account for the time-delay terms by incorporating population values from earlier time steps into the difference equation system. In other words, the difference equations for populations at time 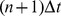 may depend not only on population values from the immediately preceding time step 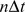, but also on population values from earlier time steps 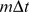 for 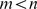. The system of difference equations that we obtain is given below.

Let 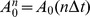, 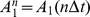, 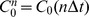, 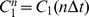, and 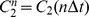. Then we rewrite (3) as the following analogous difference equation system:
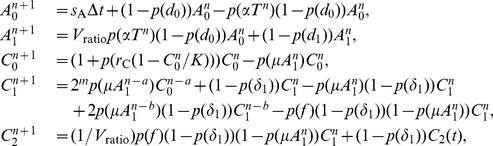
(4)where 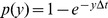, 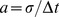, and 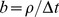. Here, we assume that 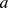 and 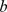 are positive integers.

The first equation in (4) pertains, as before, to APCs waiting in the periphery. The first term of the equation is the rate at which new APCs are supplied into the system during one time step 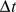. The coefficient 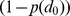 of the second term is the probability that a cell survives the next time step. Hence, the second term 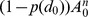 is the concentration of APCs that survive from time 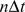 to 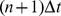. The coefficient 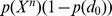 of the third term is the probability that an APC survives the next time step and is stimulated to become a mature, antigen-bearing APC. The coefficients in the the first three equations in (4) similarly express other transition probabilities.

As before, the fourth equation in (4) pertains to effector CTLs in the lymph node. The first term is the rate at which activated memory CTLs enter the effector state after completing 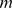 divisions. As in (3), this term depends on mature APC and memory CTL concentrations 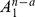 and 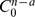 from 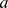 time steps earlier. The factor 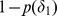 in the second term is the probability that a cell survives the next time step. The factor 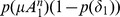 in the third term is the probability that a cell survives the next time step and gets stimulated by a mature APC to undergo further division. The fourth term is the rate at which dividing CTLs reenter the system 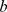 time steps later. The factor 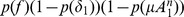 in the final term is the probability that a cell survives the next time step, does not get stimulated to divide, and flows out of the lymph node to the periphery. The terms in the final equation of (4) are similar to those already discussed.

The advantages of rewriting the DDEs (3) as the difference equations (4) are that the difference equation can be updated in parallel with the ABM with time steps of length 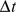, rewriting transition rates from the DDEs in terms of probabilities for the difference equations is consistent with the probabilistic treatment of cell behavior in the ABM, and the numerical values of the difference equations are guaranteed to remain nonnegative. Since the time step 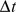 that we use is relatively small, numerical solutions of (3) using the Matlab function ‘dde23’ and numerical evaluations of (4) are nearly indistinguishable. Using the ABM algorithm and the difference equation system (4), we simulate the combined system in the following steps:

1. At time 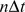, record the current total tumor cell population, 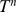, from the ABM and the current CTL concentration, 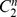, from the difference equation system. These values are used as inputs into the difference equation system and the ABM, respectively.
2. Simulate one time step of the ABM and difference equation system.
3. Repeat from step 1 for the next time step.

### Parameter estimates

Parameter estimates for the ABM are shown in Table 1. We discuss how we obtained the estimates below.

For our simulations, we set the time step to 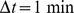, because 1 minute is the timescale of the fastest dynamic simulated in the model, i.e. CTL motion. For the cell radius, we estimate 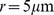, since typical diameters of CTLs and tumor cells fall around 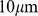 [12], [21], [24], [46].

By fitting a growth model to experimental breast tumor data, Spratt *et al.* estimate that the initial tumor cell doubling time is between 30 and 4800 days (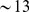 years) [28]. In another study, Weedon-Fekjær *et al.* obtain similar doubling times of 1.2 months to 6.3 years [29]. Other experimental studies report long-term doubling times of around 100 days [26], [27]. However, some mathematical models consider the possibility of aggressive early-stage tumors with division times of under 10 days [8], [24]. To model a relatively fast-growing tumor, we estimate the tumor division time, 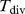, to be 7 days, but consider a range from 1 to 400 days. As we see in the Results, this range is sufficient to clarify how this parameter influences the model.

We model CTL motion using a Wiener process, so it is difficult to speak of velocity. Instead, we use the standard deviation of distance displaced per unit time as a substitute measure. Friedl and Gunzer estimate that CTLs migrate at mean velocities of 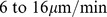, and Catron *et al.* choose an estimate of 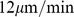 [35], [46]. Therefore, we set the maximum unit standard deviation, 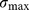, of CTL motion to be 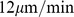, but consider a range from 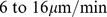.

It is difficult to estimate how long it takes a CTL to accelerate from stationary to its maximum diffusion rate, so we suppose that the acceleration time, 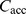, takes approximately 5 hours and consider a wide range from 0 to 24 hours.

The experimentally measured half-life of effector CTLs during contraction is 41 hours, so in the model, we set 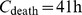 [30]. We do not have clear estimates of the average times for CTL recruitment, 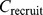, and CTL killing, 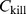. However, experimental studies show that anti-tumor CTLs can effectively recruit additional CTLs [3], [4] and rapidly kill target cells, sometimes even killing multiple target cells simultaneously [49]. To consider a wide range, we assume that the average CTL recruitment time, 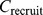, is 8 hours, but we consider a range of 2 to 24 hours. Since killing target cells may require a a long recovery period, we assume that the average CTL killing time, 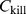, is 24 hours, but we consider a range of 4 to 48 hours. We consider this an adequate range since the CTL half-life is 41 hours. We do not consider rates of 0 hours, because that would mean CTLs can recruit or kill infinitely fast.

The requirement for the region of interest is that it is large enough to contain the relevant tumor-immune dynamics without inducing too many effects from dynamics occurring too close to the boundary. As a result, we set the radius of the region of interest to be 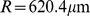, since such a region can adequately simulate a spherical tumor of over 50,000 cells with ample surrounding space, and the volume of the region conveniently comes out to 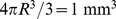.

Similarly, we require the CTL cloud to be wide enough that any CTLs that may be beyond the cloud have a very low chance of migrating across the cloud and into the region of interest during one time step. Since CTLs move according to a Wiener process with unit standard deviation 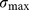, the distance a CTL will move orthogonally toward the surface of the region of interest in one time step is given by the normal distribution 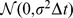. Hence, if we set the width of the CTL cloud to be 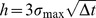, the probability that a CTL could pass from outside the cloud into the region of interest is 0.001.

A list of parameters with estimated values for the DDE is shown in Table 1. We discuss how we obtained the estimates below.

An experimental study measuring the volumes of head and neck lymph nodes in men and women estimate lymph node volumes ranging from 0.1 to 1 mL, depending on the location of the lymph node [50]. If we assume our lymph node compartment is approximately 1 mL and that the breast tissue is approximately 1 L, we obtain a volume ratio of 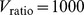.

Cell concentrations are obtained from a study by Catron *et al.* in which they simulated a hypothetical, spherical, skin-draining lymph node of radius 1 mm [46]. In their paper, they considered a slice of about 1/500 of the total volume and estimated that the slice contains about 1600 CTLs (CD8+ T cells) and 100 dendritic cells (DCs) [46]. Such a slice would have a volume of 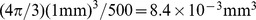, yielding T cell and DC concentrations of approximately 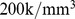 and 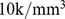, respectively.

We assume that the lymph node contains a population anti-tumor memory CTLs, which were previously induced by a preventative anti-tumor vaccine. For a base estimate, we assume that the equilibrium memory CTL concentration, 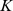, in the lymph node is 2% of 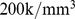, and we consider a range of 1 to 10% of 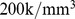. Since we are setting initial conditions for DDEs, we are interested in the history of cell concentrations on the time interval 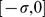, so we assume that the system was at steady state before time 0 and set 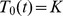 for 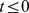. For the logistic growth rate, we estimate that memory CTLs replenish at rate 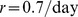, which corresponds to a minimum doubling time of 1 day. As seen in the Results, only a very small fraction (less than 1%) of memory CTLs becomes activated by the incipient tumor, so variations in the replenishment rate of memory CTLs does not significantly influence the outcome of the simulations (results not shown), so we do not consider it worthwhile to vary this parameter along with the others.

Since DCs are the primary APCs that stimulate T cells [6, p. 319], we assume that our estimate of the DC concentration is also a good estimate of the APC concentration. We do not know how many APCs reside in a tissue that drains into a particular lymph node, but we assume that it is of the same order of magnitude as the number of APCs in the lymph node. Hence, we estimate that the initial concentration of APCs in the tissue before time 0 is 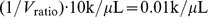, i.e., 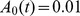 for 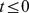. We assume that all other cell concentrations start at 0.

Next, we estimate the death and supply rates of immature APCs. Since we are dealing with a closed system, we recognize that cells may leave the system due to random circulation or emigration, but for convenience, we incorporate these cases into the death rates. Not having explicit references for the turnover rates of immature APCs in tissue, we assume they are similar to those of naïve T cells, which is estimated to be around 3% per day [51]. Hence, we set the immature APC death rate, 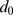, to be 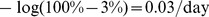 and calculate the steady state supply rate to be 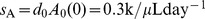.

The half-life during T cell contraction is 41 h, so we estimate an effector CTL death rate of 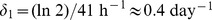 [30]. Furthermore, the level of antigen presentation following the third day after infection decays with a half-life of around 19 h and 20.4 h [52]. Hence, using a half-life of 20 h, we obtain a mature APC death rate of 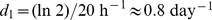. We note that these APCs might not actually be dying. Instead, they might be turning over surface molecules, but for our purposes, these APCs can be considered eliminated.

For the minimal CTL division program, various studies estimate that newly activated CTLs (from a naïve state) undergo between 7 and 10 initial divisions [36], [53], and that a responding CTL population could expand up to five orders of magnitude [34]. This range corresponds to between 7 and 17 cell divisions. Since activated memory CTLs probably undergo more divisions than newly activated naïve CTLs, we assume a base estimate of 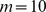 divisions upon activation and consider a range from 7 to 17 divisions.

To calculate the mass-action coefficient, 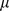, we use the estimate that in the lymph node slice of Catron *et al.*, one T cell and one DC will have 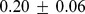 interactions per hour, or 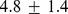 interactions per day [46]. Assuming that DCs represent the majority of APCs that stimulate T cells, we obtain an estimate of the mass-action coefficient 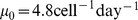 [46]. Recalling that the lymph node slice has a volume of 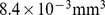, we obtain the unit conversion
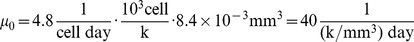
It is unlikely that every antigen-specific CTL-APC interaction leads to CTL stimulation, so we set the probability of successful stimulation to 0.5 as a base estimate and consider probabilities from 0.05 to 0.5. These estimates translate to a base estimate of 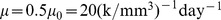 for the mass-action coefficient and a range of 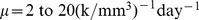.

For the time delays, the duration of one division is between 6 to 12 hours (i.e., 2 to 4 times per day) [6, p. 19]. In addition, the T cell population doubles approximately every 8 hours during expansion [30]. We use the intermediate value of 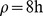, or 1/3 day, as a base estimate and consider a range from 4 to 24 hours. The CTL division program consists of 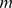 divisions, but the first division does not occur until 24 hours after stimulation [54], [55]. Hence, we set the duration of the division program to be 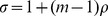 to account for the fact that the first division takes one day while subsequent divisions take 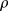 days.

We do not have a good estimate of the antigenicity, 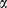, of the tumor, so we assume a base value of 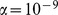 and consider a range from 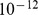 to 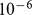. The parameter 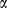 can be understood to represent the reciprocal of the rate at which one APCs will encounter and take up antigen from one tumor cell in the tissue. In other words, if we assume that the APC concentration is 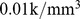 (i.e., 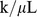) in the tissue and that the tissue has a volume of 1 L, then there are 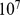 APCs circulating in the tissue. As a result, if 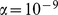, it will take an average of 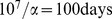 for one circulating APC to encounter antigen from a single tumor cell in the tissue. A range of 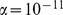 to 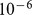 corresponds to average discovery times of a single APC from 10,000 days (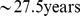) to a couple of hours.

For the flow rate of effector CTLs out of the lymph node to the tissue, we assume that effector CTLs that are not being stimulated to divide emigrate from the lymph node at a half life of 1 day, so that the flow rate 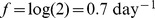.

### CTL acceleration

To derive (2), we take advantage of the connection between random walks on a lattice and the Wiener process. Suppose that at each time step 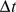, a CTL has equal probability of moving distance 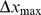 in any of the six cardinal directions on a 3-D square lattice. If we let 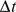 and 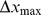 go to zero in such a way that 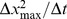 remains constant, the random walk approaches a Wiener process corresponding to a diffusion rate 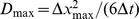 and unit standard deviation 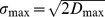 [41], [42].

Suppose a stationary CTL accelerates at a constant rate and reaches the maximum velocity at time 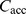. Hence, at time 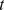 after beginning acceleration, the CTL has a velocity that is 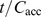 of the maximum, so we suppose that the CTL conducts a random walk of steps size 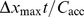 instead of the maximum step size. The associated diffusion rate for this random walk is 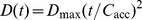, which yields a unit standard deviation of 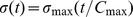. Since a CTL's motion cannot exceed the maximum rate given by 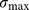, we obtain the expression given in (2).
